# Sigma-1 Receptor Positron Emission Tomography: A New Molecular Imaging Approach Using (*S*)-(−)-[^18^F]Fluspidine in Glioblastoma

**DOI:** 10.3390/molecules25092170

**Published:** 2020-05-06

**Authors:** Magali Toussaint, Winnie Deuther-Conrad, Mathias Kranz, Steffen Fischer, Friedrich-Alexander Ludwig, Tareq A. Juratli, Marianne Patt, Bernhard Wünsch, Gabriele Schackert, Osama Sabri, Peter Brust

**Affiliations:** 1 Helmholtz-Zentrum Dresden-Rossendorf (HZDR), Institute of Radiopharmaceutical Cancer Research, Department of Neuroradiopharmaceuticals, Research site Leipzig, 04318 Leipzig, Germany; w.deuther-conrad@hzdr.de (W.D.-C.); mathias.kranz@uit.no (M.K.); s.fischer@hzdr.de (S.F.); f.ludwig@hzdr.de (F.-A.L.); p.brust@hzdr.de (P.B.); 2 PET Imaging Center, University Hospital of North Norway (UNN), 9009 Tromsø, Norway; 3 Nuclear Medicine and Radiation Biology Research Group, The Arctic University of Norway, 9009 Tromsø, Norway; 4 Department of Neurosurgery, Technische Universität Dresden (TUD), University Hospital Carl Gustav Carus, 01307 Dresden, Germany; tareq.juratli@uniklinikum-dresden.de (T.A.J.); gabriele.schackert@uniklinikum-dresden.de (G.S.); 5 Department of Nuclear Medicine, University Hospital Leipzig, 04318 Leipzig, Germany; marianne.patt@medizin.uni-leipzig.de (M.P.); osama.sabri@medizin.uni-leipzig.de (O.S.); 6 Institute of Pharmaceutical and Medicinal Chemistry, University of Münster, 48149 Münster, Germany; wuensch@uni-muenster.de

**Keywords:** sigma-1 receptor availability, orthotopic xenograft of glioblastoma in mouse, small animal Positron Emission Tomography/Magnetic Resonance Imaging (PET/MRI), (*S*)-(−)-[^18^F]fluspidine, imaging-based biomarker

## Abstract

Glioblastoma multiforme (GBM) is the most devastating primary brain tumour characterised by infiltrative growth and resistance to therapies. According to recent research, the sigma-1 receptor (sig1R), an endoplasmic reticulum chaperone protein, is involved in signaling pathways assumed to control the proliferation of cancer cells and thus could serve as candidate for molecular characterisation of GBM. To test this hypothesis, we used the clinically applied sig1R-ligand (*S*)-(−)-[^18^F]fluspidine in imaging studies in an orthotopic mouse model of GBM (U87-MG) as well as in human GBM tissue. A tumour-specific overexpression of sig1R in the U87-MG model was revealed in vitro by autoradiography. The binding parameters demonstrated target-selective binding according to identical K_D_ values in the tumour area and the contralateral side, but a higher density of sig1R in the tumour. Different kinetic profiles were observed in both areas, with a slower washout in the tumour tissue compared to the contralateral side. The translational relevance of sig1R imaging in oncology is reflected by the autoradiographic detection of tumour-specific expression of sig1R in samples obtained from patients with glioblastoma. Thus, the herein presented data support further research on sig1R in neuro-oncology.

## 1. Introduction

Glioblastoma multiforme (GBM) is the most common primary tumour of the central nervous system. Although the global incidence is rare with less than 10 per 100,000 people, the median survival rates for patients with GBM remain dramatically low despite complex surgical, pharmacological and radiation therapy approaches [1,2]. An important aspect contributing to this poor outcome is the genetic heterogeneity of GBM, which translates into heterogeneous expression patterns of potentially druggable targets [3]. Accordingly, the development of new targeted therapies as well as of biomarkers for predictions of treatment response would benefit from an improved understanding of how such spatiotemporal patterns evolve and change during pathogenesis [4,5,6,7,8,9]. Nuclear medicine imaging techniques offer a unique possibility to noninvasively assess the distribution and amount of certain biological targets and thus to contribute significantly to the drug-discovery process and later on to the evaluation of the treatment efficacy [10,11,12].

By application of suitable radiolabeled molecules, positron emission tomography (PET) in particular can assess such alterations with high sensitivity. Imaging agents for the investigation of the catabolic and anabolic metabolism can detect cancer-specific alterations in high-capacity processes such as glycolysis (by [^18^F]FDG), amino acid transport (by [^11^C]MET or [^18^F]FET), and membrane turnover (by [^18^F]FMC) [13,14]. They are currently utilized to improve the clinical management of brain cancer patients. Furthermore, the PET technology offers the principal possibility to investigate differences in the expression pattern and activity of diagnostically and therapeutically relevant proteins, such as receptors or enzymes, and to correlate them with tumour heterogeneity and aggressiveness. The current development of radiolabelled probes to image e.g., isocitrate dehydrogenase mutations (IDH1R132H) [15], or the glutamate carboxypeptidase II (prostate-specific membrane antigen, PSMA) [16], reflects the interest in preclinical and clinical research on detailed and targeted molecular characterisation of malignancies in the brain, which is a prerequisite to define the role of nuclear medicine imaging for the individualized treatment of patients with GBM [14,17].

Our research on the identification of new targets for brain cancer imaging focuses on the sigma-1 receptor (sig1R), an intracellular chaperone protein highly expressed in a variety of cancers including GBM [18,19]. Under physiological conditions, the sig1R is localized at the mitochondrion-associated endoplasmic reticulum membrane (MAM) and at the plasma membrane and is involved by interactions with other proteins in a number of pathways related to the metabolism and proliferation of cells. Accordingly, albeit at different levels, sig1R is expressed in all peripheral organs as well as in the central nervous system. Widely distributed in the brain [20], the sig1R is involved in memory, emotional, and sensory functions and changes in its expression are related to neurodegenerative diseases such as Huntington ’s disease and Alzheimer’s disease, as well as in stroke, depression and pain disorders [20,21]. Most likely, due to the translocation of the intracellular receptor from MAM to the plasma membrane and the cell nucleus, which is triggered under pathological conditions [22,23,24,25], sig1R is functionally involved into a variety of cellular pathways related to stress response and survival [25,26,27]. In addition, the expression of sig1R seems to be upregulated by cancer-specific mechanisms, as indicated by the high levels of sig1R protein discovered in many cancer cell lines [19,26,27,28]. The antiproliferative effect of pharmacological inhibition of sig1R by putative antagonistic ligands on cancer cell lines further substantiates the potential role of sig1R in cancer biology [27,29,30,31,32,33]. Sig1R ligands influence apoptosis, migration, and cell cycle progression pathways through their interaction with voltage-dependent K^+^ channels, volume-regulated Cl^−^ channels, or endoplasmic reticulum Ca^2+^ release [22,31,32,34]. Altogether, the available data present strong evidence of an important role of sig1R in tumour biology, and for that reason, PET noninvasive molecular imaging of sig1R is assumed to improve our understanding of the role of this particular protein in tumour pathophysiology and to promote the development of a sig1R-targeted-therapies [35].

Already different radiotracers have been developed to investigate the expression of sig1R by PET such as [^18^F]FMSA4503 [36], [^18^F]SFE or [^18^F]FTC-146 [37,38]. However, only [^11^C]SA4503 and (S)-(−)-[^18^F]fluspidine were applied in research on the in vivo imaging of sig1R in brain tumours using heterotopic brain tumour models, as by the group of van Waarde, or orthotopic models, as by our group [39,40].

Encouraged by the approval of the in-house developed radiopharmaceutical (S)-(−)-[^18^F]fluspidine for clinical trials (EudraCT Numbers: 2014-005427-27, 2016-001757-41), the promising results of a collaborative pilot study in an orthotopic mouse model of GBM [40], and the establishment of orthotopic brain tumour models in our group, we decided to investigate further the relevance of sig1R in brain cancer biology and to evaluate the potential of (S)-(−)-[^18^F]fluspidine-PET to characterise brain tumours on a molecular level. We report herein on the assessment of GBM-specific expression of sig1R by a combination of in vitro and in vivo approaches using mice bearing intracranial U87-MG tumours as a preclinical orthotopic model of human GBM and the sig1R-specific radioligand (S)-(−)-[^18^F]fluspidine. Initially, we validated the in vivo selectivity of (S)-(−)-[^18^F]fluspidine for sig1R in a sig1R-knockout mouse model. Then, we validated by radioligand binding assays the suitability of the U87-MG cell line used for the orthotopic GBM model regarding the presence of the target, and confirmed by autoradiography the unimpaired overexpression under in vivo conditions. By immunohistochemistry as radioligand-independent method, we could confirm the expression and overexpression of sig1R protein in U87-MG cells in 2D culture as well as in the cellular environment of the mouse brain. Finally, we performed dynamic PET/MRI (magnetic resonance imaging) studies to assess the pharmacokinetics of (S)-(−)-[^18^F]fluspidine in the orthotopic U87-MG mouse model of human GBM, and report on the very first detection of sig1R protein in human GBM tissue by means of in vitro autoradiography, validating the relevance of this target.

## 2. Results

### 2.1. Expression of sig1R in U87-MG Cells

#### 2.1.1. Expression of sig1R in U87-MG Cells in 2D Cell Culture

We initially evaluated the expression of sig1R in U87-MG cells, a human primary glioblastoma cell line, grown in 2D cell culture by radioligand binding assays and immunohistochemistry to determine their suitability for the intended orthotopic mouse model of GBM. By a single saturation assay using (+)-[^3^H]pentazocine, an established sig1R-specific radioligand, a *B*_max_ value of 129 fmol/mg protein and a *K*_D_ value of 2.4 nM was determined. Specific binding of (S)-(−)-fluspidine towards (+)-[^3^H]pentazocine-labeled binding sites in U87-MG cells has been proven by displacement studies, and the affinity of (S)-(−)-[^18^F]fluspidine to sig1R has been determined with a *K*_D_ of 16.7 nM.

**Figure 1:**
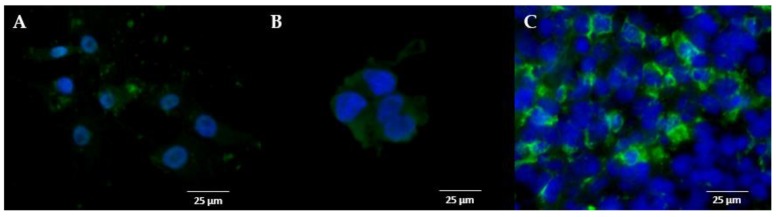
Immunofluorescent staining of sigma-1 receptors (sig1R). Representative image of the sig1R staining (**A**) in U87-MG cells grew in vitro, (**B**) in HEK-293 cells overexpressing human sigma-1 receptor (hsig1R) grew in vitro and (**C**) in a cryosection of U87-MG tumour cells orthotopically implanted in a mouse brain (scale bar: 25 µm, x40, green channel: sig1R staining, blue channel: nucleus staining).

To verify the identity of the specific binding site of the two radioligands by an independent method, we further performed immunohistochemistry using a sig1R-specific antibody. The thereby determined cytoplasmic staining of protein in isolated U87-MG cells, which corresponds with the labelling of sig1R in the positive control HEK-293 cells overexpressing human sig1R, is demonstrated in Figure 1A, B, respectively.

#### 2.1.2. Expression of sig1R in U87-MG Cells Grown In Vivo 

Because transplantation of human cancer cells into mice might be associated with an altered expression profile due to the significant change in the microenvironment, we subsequently investigated orthotopically implanted U87-MG tumour cells by immunohistochemistry and radioligand binding studies.

The strong immunofluorescence signal determined in tumour cells in cryosections obtained from mouse brain at day 27 after intracerebral transplantation of U87-MG cells indicates the persistently high expression of sig1R in the orthotopic GBM (Figure 1C). Besides, the fluorescent staining is informative in that not all cells in the field of view express sig1R, indicating a heterogeneous expression profile within the tumour bulk (Figure 1C). Thus, the conservation of sig1R expression irrespective of the environment (culture medium or brain dynamic environment) was confirmed (Figure 1).

**Figure 2:**
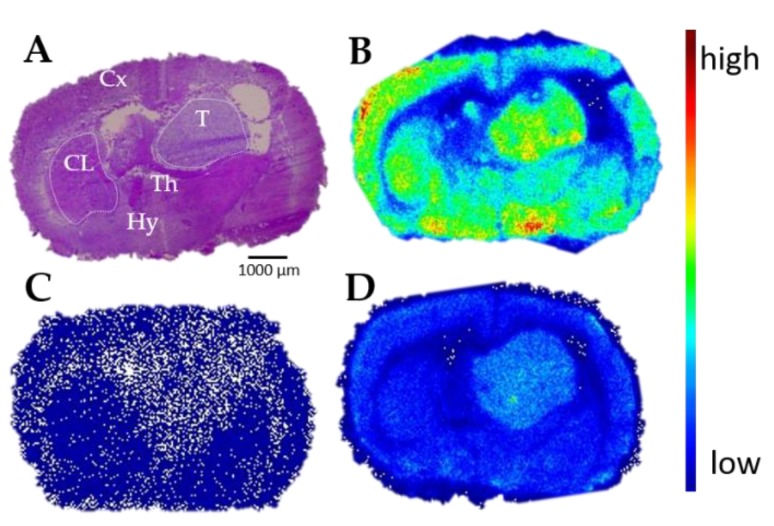
In vitro autoradiography of the mouse brain bearing an orthotopic U87-MG xenograft. Representative autoradiographic images of the coronal plane of mouse brain slices: (**A**) Hematoxylin-eosin staining; (**B**) in vitro distribution of activity after incubation with 0.1 MBq/mL (S)-(−)-[^18^F]fluspidine, (**C**) co-incubation with 10 µM SA4503 to determine the nonspecific binding and (**D**) with 10 nM of (S)-(−)-fluspidine as competing agent. Cx: cortex; CL: contralateral striatum; Th: thalamus; Hy: hypothalamus; T: tumour. Width of a mouse brain ~1 cm.

Complementary autoradiography performed with the sig1R-specific PET tracer (S)-(−)-[^18^F]fluspidine confirmed the expression of sig1R in orthotopically growing U87-MG cells. The autoradiographic images presented in Figure 2 indicate a high density of binding sites in the tumour region (Figure 2B). The radioactive signal is nearly completely abolished by co-administration of the sig1R-specific ligand SA4503, a selective agonist commonly used as competitive agent (IC_50_ = 17.4 nM [41]) (Figure 2C). Interestingly, the macroscopic distribution pattern of (S)-(−)-[^18^F]fluspidine in the orthotopic tumour reflects a heterogeneous accumulation of activity within the tumour, similar to what was observed by immunohistochemistry on cellular level. By saturation studies, performed as homogenous radioligand displacement experiments by co-incubation of (S)-(−)-[^18^F]fluspidine with different concentrations of (S)-(−)-fluspidine, we determined the kinetic binding parameters of (S)-(−)-[^18^F]fluspidine in the tumour (T) and an internal reference region, the contralateral striatum (CL). In both regions, (S)-(−)-[^18^F]fluspidine bound specifically and with comparable affinities of *K*_D_, _T_ = 17.5 ± 1.3 nM and *K*_D, CL_ = 17.0 ± 4.8 nM. However, the sig1R density was ~1.7 times higher in the tumour area compared to the CL area, as reflected by values of *B*_max, T_ = 704 ± 16 fmol/mg protein vs. *B*_max, CL_ = 414 ± 36 fmol/mg protein.

### 2.2. Assessment of GBM-Specific (S)-(−)-[^18^F]Fluspidine Kinetics In Vivo by Dynamic Small-Animal PET Imaging Studies

#### 2.2.1. Additional Approval of Target Specificity of (*S*)-(−)-[^18^F]Fluspidine PET

**Figure 3:**
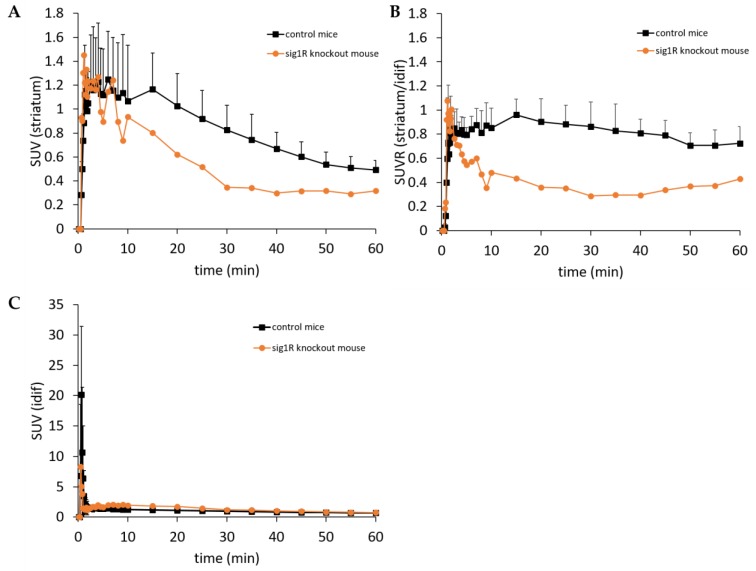
Positron Emission Tomography/ Magnetic Resonance (PET/MR) imaging of control mice (*n* = 3) and of sig1R-knockout mouse (*n* = 1) after i.v. administration of (*S*)-(−)-[^18^F]fluspidine. (**A**) Average striatal time-activity curves for control mice (black squares) and sig1R-knockout mouse (orange dots). (**B**) Average time-varying SUVRs of the striatum over the blood (defined from the image-derived input function (idif)) of control mice (black squares) and sig1R-knockout mouse (orange dots) (**C**) Average time-varying SUV of the blood (defined from the idif) of control mice (black squares) and sig1R-knockout mouse (orange dots).

Due to access to a limited number of sig1R-knockout mice [42], we performed a single head-to-head dynamic PET study with (*S*)-(−)-[^18^F]fluspidine in sig1R-knockout (*n* = 1) and control (*n* = 3) mice with the main focus on the tracer kinetics in the striatum. Mean injected activity was 3.5 MBq and 1.9 MBq with a molar activity of 24 GBq/µmol and 21 GBq/µmol at the time of injection, resulting in a mean chemical concentration of 4.7 nmol/kg and 3.2 nmol/kg in the control group and the sig1R-knockout mouse respectively. The corresponding time-activity curves (TACs) are presented in Figure 3. Both the sig1R-knockout and the control animals showed a rapid uptake of activity within the first minutes after i.v. injection of (*S*)-(−)-[^18^F]fluspidine (initial peak between 1 and 2 min p.i. of 1.45 and 1.12 SUV, respectively) along with a much stronger and faster washout observed in the sig1R-knockout mouse in comparison to the control animals (SUV at 10, 20, and 40 min p.i. of 0.84, 0.64, and 0.25 vs. 1.07, 1.03, and 0.67, respectively) confirming the selectivity in vivo of (*S*)-(−)-[^18^F]fluspidine for sig1R (Figure 3A). In order to correct for potential model-related differences in the brain perfusion, we calculated an SUV ratio (SUVR) from the TAC data obtained in striatum and blood at each time points. The corresponding SUVR curves, presented in Figure 3B, indicate for both animal models nearly stable SUVR values at 15 to 60 min p.i., albeit with clearly different values. While for the control animals SUVR values in the range of 0.8 to 1 (corresponding to an area under the curve (AUC) value of 48.6 ± 8.3) has been estimated, the notably lower value of about 0.4 (corresponding to an AUC value of 23.4) determined in the single sig1R-knockout mouse confirms the target specificity of (*S*)-(−)-[^18^F]fluspidine.

#### 2.2.2. GBM-Specific Pharmacokinetics of (*S*)-(−)-[^18^F]Fluspidine in the Orthotopic U87-MG Mouse Model

Encouraged by the in vitro and in vivo findings on the specific expression of sig1R in orthotopically grown U87-MG tumours and the sig1R-specific binding of (*S*)-(−)-[^18^F]fluspidine, we proceeded with PET studies with (*S*)-(−)-[^18^F]fluspidine performed under baseline conditions in nude mice-bearing orthotopic U87-MG (n = 3). Mean injected activity was 9.1 MBq with a molar activity of 92.5 GBq/µmol at the time of injection, resulting in a mean chemical concentration of 4.2 nmol/kg. The tumour growth was assessed by MRI with a T2-weighted sequence, and a 60 min dynamic PET scan, followed by T1- and T2-weighted sequences, performed when the tumour size was 28 ± 8 mm^3^ (i.e., 23 to 30 days after implantation). The regions-of-interest (ROIs) were delineated on the T2-weighted MR images and then applied on the PET data to generate the regional TACs.

**Figure 4:**
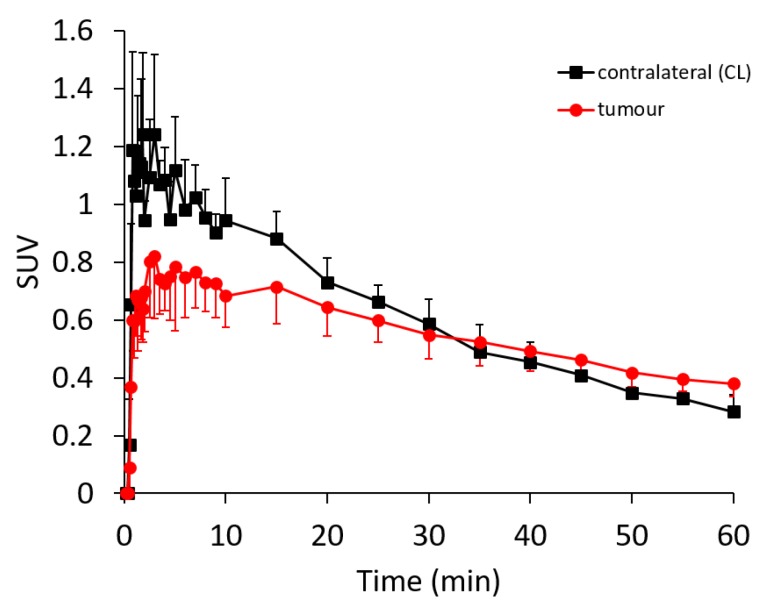
PET/MR imaging of sig1R in mice with orthotopic xenograft of human GBM cells (U87-MG). Average time-activity curves after i.v. administration of (S)-(−)-[^18^F]fluspidine of the tumour (red dots) and the contralateral (black squares) regions of interest (*n* = 3). Statistical test: Student *t*-test, * *p* < 0.05.

As reflected by the TACs presented in Figure 4, although not statistically significant, the uptake of (*S*)-(−)-[^18^F]fluspidine in the tumour is lower and more slowly than the activity uptake in the control region with maximal SUV values of 0.82 at 3 min p.i. and 1.24 at 1 min p.i., respectively. However, because the washout from the tumour was slower, the tumour and CL TACs intersected at about 30 min p.i., demonstrating with SUVs of 0.38 and 0.28 at 60 min p.i. a higher retention of activity in the tumour region compared to the CL, respectively. The retarded washout of the sig1R-specific (*S*)-(−)-[^18^F]fluspidine from the orthotopic tumour is in accordance with the autoradiographic data, indicating a higher availability of sig1R in the U87-MG tumour tissue in comparison to CL.

**Figure 5:**
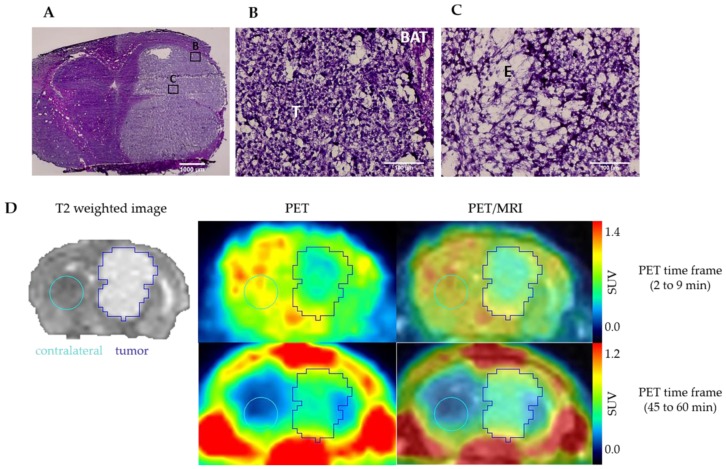
Hematoxylin-eosin staining of U87-MG tumour: (**A**) tumour bulk in the right striatum of a mouse brain (×2, scale bar: 1000 µm); (**B**) tumour periphery presents area of high density of cell nuclei; (**C**) tumour centre presents area of lower cell density accompanied by oedema. (×40, BAT: brain adjacent to tumour; T: tumour, E: oedema. Scale bar: 100 µm. (**D**) Representatives coronal PET/MR images of U87-MG tumour-bearing mouse after i.v. administration of (S)-(−)-[^18^F]fluspidine. The upper panel exhibits the distribution of (*S*)-(−)-[^18^F]fluspidine at early times p.i. (averaged time frames from 2 to 9 min), and the lower panel exhibits the distribution of (*S*)-(−)-[^18^F]fluspidine at later times (averaged time frames from 45 to 60 min).

The intratumoral heterogeneity of sig1R expression already discovered by the radioligand and antibody investigations in vitro was detectable also by the in vivo imaging study. The early PET images between 2 and 9 min after injection show an heterogeneous uptake of (*S*)-(−)-[^18^F]fluspidine into the tumour (Figure 5D, upper panel). According to the histological analyses of the explanted tumour tissue, performed immediately after the PET scans, the tumour inner part is characterised by a lower cell density compared to the periphery along with extra-cellular oedema area highlighting presumably areas of necrosis (Figure 5A–C). Therefore, the heterogeneous uptake of (*S*)-(−)-[^18^F]fluspidine may also (or additionally) be caused by reduced blood supply to the tumour centre. The PET image at later time points (45 to -60 min p.i.; Figure 5D, lower panel) pictures a more homogenous uptake of the tracer, along with a low slope, reflecting an accumulation.

### 2.3. Presence of (S)-(−)-[^18^F]Fluspidine Binding Sites in Human GBM Tissue

**Figure 6:**
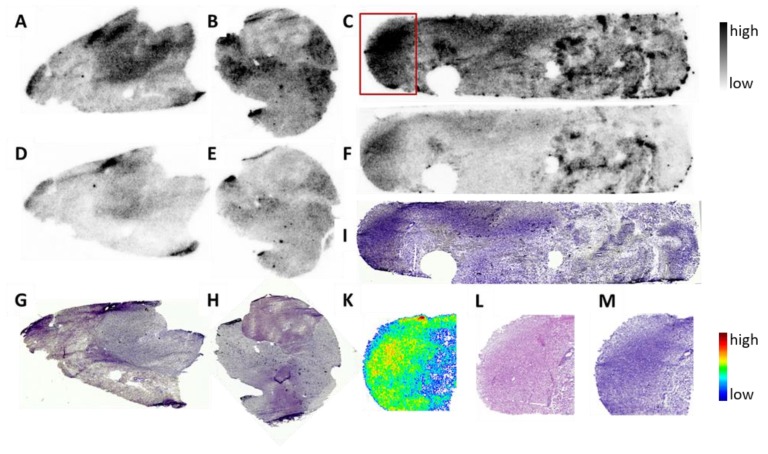
Sig1R autoradiography with the sig1R-specific PET ligand (*S*)-(−)-[^18^F]fluspidine in human GBM in vitro. Binding of (*S*)-(−)-[^18^F]fluspidine at 4.5 nM in cryosections (12 µm) of tumour tissue obtained from three patients (**A**,**B**,**C**) demonstrated heterogeneous distribution throughout the slices. By co-incubation with 1 µM haloperidol (**D,E,F**), a substantial reduction in activity accumulation was obtained. Histochemical analysis of corresponding sections was performed by Nissl staining (**G**,**H**,**I**). Analysis of one sample at higher magnification (red square in **C**) demonstrated correlation of the activity accumulation (**K**) with highly cell dense regions (H&E staining: (**L**); Nissl staining: (**M**). Length of the biopsies samples ~1 cm.

To initially assess the suitability of sig1R as specific target for molecular characterisation of human GBM, we performed in vitro autoradiography with (*S*)-(−)-[^18^F]fluspidine using cryosections of tissue samples obtained from 3 patients diagnosed with Glioblastoma multiforme IV. Total and nonspecific binding of the PET ligand was determined by incubation with only (*S*)-(−)-[^18^F]fluspidine or with co-administration of a high concentration of haloperidol to block the sig1R, followed by histological staining of the respective cryosections. As shown in Figure 6, the autoradiographic images indicate a heterogeneous pattern of binding sites of (*S*)-(−)-[^18^F]fluspidine in all three GBM samples with the highest density in regions histologically characterised by a high density of cells which we assume might be related to the highly proliferating tumour cells. Accordingly, although it was not possible within this preliminary study to confirm by immunohistochemistry the distribution pattern of sig1R in the cryosections or to identify the type of cells possessing high specific binding of (*S*)-(−)-[^18^F]fluspidine, these preliminary data motivate us to design a complementary study on the investigation of sig1R protein in a larger number of GBM samples by means of specific radioligands and antibodies.

## 3. Discussion

In this study, we evaluated the availability of sig1R in an orthotopic mouse model of human GBM. High expression of sig1R in different cancer cell lines derived from prostate, breast, colon, melanoma, small and non-small cell lung cancer, brain tumours including GBM, neuroblastoma, and meningioma have already been reported [18,19,26,27,43,44,45,46,47,48]. The involvement of sig1R in many but selective protein interactions, the antiproliferative effect of putative antagonists, as well as their nonpleiotropic effects make sig1R a potent drug target prone to overcome adaptative drug resistance alone or in combination with other drugs [49,50]. It is also known that the upregulation of sig1R on both mRNA and protein level in the same cancer subtype differs from one cell line to another and from one patient to another, probably reflecting a context-dependent expression of sig1R [51,52,53]. Consequently, an improved understanding of how such patterns evolve and change during pathogenesis, by the use of noninvasive PET imaging, would promote the development of sig1R-based therapies. In this context, we chose to evaluate by PET the suitability of the clinically approved imaging agent (*S*)-(−)-[^18^F]fluspidine for the analysis of the expression of sig1R in GBM.

We first investigated in vitro the level of expression of sig1R protein in U87-MG cells, a human GBM cell line widely applied for orthotopic brain cancer mouse models. As consistently reported, sig1R is located at the endoplasmic reticulum-mitochondria interface and redistributes ligand-mediated and under conditions of cellular stress dynamically to the plasma membrane and the nucleus envelope [54,55]. The examination of sig1R expression in U87-MG cells by using (+)-[^3^H]pentazocine, a selective sig1R ligand widely applied in radioligand binding assays [56,57], demonstrated high affinity binding towards a single binding site expressed in U87-MG cells grown in 2D cell culture. The density of sig1R in this cell line, *B*_max_ = 129 fmol/mg protein, is in the range of values determined by (+)-[^3^H]pentazocine in other cancer cell lines such as 42 fmol/mg protein in the C6 murine glioblastoma cells, 76.5 fmol/mg protein in the NB41A3 neuroblastoma cells or 1115 fmol/mg protein in the U-138-MG cells [19]. We identified the U87-MG cells as suitable for the orthotopic GBM model applied in this study. 

As the microenvironment is known to influence gene expression, e.g., the hypoxia-stimulated HIF-1α expression in glioma or the culture mode (2D vs. 3D)-dependent differential gene expression of colorectal cell lines [58,59,60,61], we further investigated the expression of sig1R in the intracerebral U87-MG tumour. The comparable immunofluorescence staining on 2D-cultivated U87-MG cells and on the orthotopically grown U87-MG tumour along with the similarity of the K_D_ values of the PET tracer (S)-(−)-[^18^F]fluspidine, indicate the conservation of sig1R expression and conformation over the translation from in vitro culture to in vivo implantation. Furthermore, the cytoplasmic localization of the sig1R fluorescence signal which was observed in vitro and in the explanted brain tumours matches with the cellular localization of the receptor found in rat astrocytes and mouse neurons [62,63].

Subsequently, we quantified the number of sig1R expressed in the U87-MG tumours implanted in the right striatum as well as in the internal control region, the left striatum. The equivalent *K*_D_ values obtained for (*S*)-(−)-[^18^F]fluspidine in both regions indicate that the PET radiotracer binds to the same target, i.e., the sig1R, in both compartments. The analysis of the binding parameter *B*_max_ excludes conformational differences between sig1R in cancer and normal cells, as discussed by Kim et al., as a possible reason for the higher accumulation of sig1R-targeting radioligands in tumour tissue, but clearly indicates an about 2-fold higher density of sig1R in the U87-MG tumour in comparison to the healthy brain [51]. Thus, the herein exploited orthotopic U87-MG GBM mouse model is appropriate for the following imaging studies. As an add-on to the extensive and validated data on the selectivity of the clinically applied PET radioligand (*S*)-(−)-[^18^F]fluspidine obtained mainly by pharmacological intervention studies [64,65], we made use of access to a sig1R-knockout mouse model to measure the actual contribution of the off-target binding of the radiotracer to the uptake of activity in the brain in imaging studies in mouse [66]. In accordance with the fast washout kinetics observed in the knockout model, we supposed only a weak background signal in imaging studies with (*S*)-(−)-[^18^F]fluspidine in the orthotopic brain cancer model and no relevant interaction with off-target binding sites in vivo.

Eventually, the results of the fundamental characterisation of the components of the experimental setting, i.e., the mouse model and the PET radioligand, with respect to availability of and selectivity to sig1R, prompted us to proceed with dynamic PET studies in the orthotopic U87-MG glioblastoma mouse model. Only few studies have explored the use of PET radiotracers for sig1R imaging of tumours, and even less have addressed brain tumours in particular [36,39,67,68,69,70] such as the investigation of sig1R in an ectopic glioma rat model as well as in spontaneous pituitary tumours in rats using [^11^C]SA4503 by the group of van Waarde [39,69,70]. To the best of our knowledge, we are the first exploring the sig1R availability of human glioblastoma in an orthotopic tumour mouse model. The in vivo imaging studies revealed a tumour-to-background ratio (TBR) of only slightly higher than 1, detectable from the late PET images. Even though we observed a continuous washout of activity from the tumour, this process was slower than in contralateral tissue. Accordingly, the activity concentration in the tumour surpassed that in the contralateral striatum over time.

Despite this, the TBR value determined in our study is in fact notably lower than the values reported for the [^11^C]SA4503 PET studies mentioned above. However, we assume that this discrepancy is related mainly to the characteristics of the background region, in particular the physiological expression of sig1R in the different grafting sites. An ectopic tumour obtained by e.g., implantation of C6 glioma in the shoulder in the soft tissue [71], close to the muscle, benefit of an ideal background tissue with low expression of sig1R [20], leading to a TBR values > 4. Such values are not comparable to orthotopically transplanted brain tumours due to the comparatively high expression of sig1R in the surrounding nondiseased brain, as indicated by e.g., in the herein performed PET studies with (*S*)-(−)-[^18^F]fluspidine in healthy mice [72,73].

The reasons for the discrepant results obtained in the present paper regarding the in vitro and in vivo quantification of sig1R in the U87-MG tumours are not clear at the moment. We assume, that factors such as microenvironment, vascularisation, or interstitial fluid pressure affect the binding parameters of (*S*)-(−)-[^18^F]fluspidine in vivo. The U87-MG tumour is known to be highly vascularised and presenting necrotic foci [62], suggesting a first uptake in the vascularised periphery and a later accumulation by diffusion in the core of the tumour tissue. However, a systematic investigation of these processes was beyond the scope of this study. Notwithstanding this limitation, a detailed investigation of the PET images of the intracranial U87-MG tumours revealed that the heterogeneous pattern of activity accumulation discovered already in vitro could be detectable by the in vivo imaging approach as well. Interestingly, a similar distribution of [^11^C]SA4503 in the tumour outer rim was reported in the already mentioned PET study of the ectopic C6 glioma model as well as in a patient with non-small cell lung cancer in the tumour tissue [74,75]; noteworthy is the discrepancy between the distribution patterns of [^11^C]SA4503 and [^18^F]FDG [69,74]. Since [^18^F]FDG PET images may be misleading due to an increased glucose metabolism in noncancerous but inflammatory tissues, the authors suggested the use of sig1R PET imaging to discriminate between tumour and inflammation [69].

A final aspect addressed in this study on the suitability of PET imaging of sig1R in glioblastoma was the investigation of the expression of sig1R in human GBM tissue. The accordingly performed receptor autoradiography with (*S*)-(−)-[^18^F]fluspidine on cryosections of human glioblastoma obtained from three patients consistently showed a heterogeneous distribution of binding sites of the sig1R-targeting radioligand with high-density binding in cell-dense regions as suggested by the subsequent histological analysis. However, although sig1R appears to play a role in proliferation, this preliminary examination does not allow to speculate about a correlation between receptor expression and tumour proliferation but nevertheless suggests to design a respective large-scale study [28].

## 4. Materials and Methods

All experimental work including animals has been conducted in accordance with the national legislation on the use of animals for research (Tierschutzgesetz (TierSchG), Tierschutz-Versuchstierverordnung (TierSchVersV)) and has been approved by the responsible research ethics committee (TVV 30/17; TVV 18/18 Landesdirektion Sachsen).

### 4.1. Radiochemistry

Enantiomerically pure (*S*)-(−)-[^18^F]fluspidine was prepared on a TRACERlab FXN synthesizer (GE Healthcare, Waukesha, WI, USA) as described in previous publications [65]. The radiochemical purity of (S)-(−)-[^18^F]fluspidine was >99%, and the molar activity (A_m_) at the end of the synthesis (EOS) was 89–180 GBq/µmol (*n* = 2).

### 4.2. Cell Culture

U87-MG cells (obtained from Jens Pietzsch/Birgit Belter, Department Radiopharmaceutical and Chemical Biology, Helmholtz-Zentrum Dresden-Rossendorf, Rossendorf, Germany) and human hsig1R-transfected Human Embryonic Kidney (HEK) cells (obtained from Olivier Soriani, Institut de Biologie Valrose—University Côte d’Azur, Sophia Antipolis, France) were maintained in monolayer culture (37 °C, 5% CO_2_, 95% O_2_) in Dulbecco’s Modified Eagle Medium (DMEM, Gibco, Invitrogen, Dun Laoghaire, Ireland) supplemented with 10% heat inactivated fetal bovine serum (Gibco, Invitrogen, Dun Laoghaire, Ireland), 5% penicillin and streptomycin, 1.25% sodium pyruvate, 1% l-glutamine (Gibco, Invitrogen, Ireland) and 1 µg/mL puromycin (Gibco, Invitrogen, Dun Laoghaire, Ireland) only for the transfected cells.

### 4.3. In Vivo Competitive Radioligand Binding Assay

Cell membrane homogenates of U87-MG cells were obtained by gentle scraping the cells grown to confluency in one 175 cm^2^ flask, followed by sedimentation of the cells suspended in cell culture medium by centrifugation at 800 rpm for 3 min at room temperature, re-suspension of the cells in 1 mL 50 mM TRIS-HCl, pH 7.4/4 °C and incubation on ice for 20 min, centrifugation of the suspension at 15,000 rpm for 15 min at 4 °C, and finally re-suspension of the pellet in 200 µL 50 mM TRIS-HCl, pH 7.4/4 °C and storage at −25 °C. The radioligand binding assay was performed by incubating the U87-MG cell membrane homogenate (226 µg protein/mL) with the Sig1R agonist (+)-[^3^H] pentazocine (working concentration = 3.25 nM; A_m_ = 995 GBq/mmol; PerkinElmer LAS GmbH, Rodgau, Germany) in incubation buffer (50 mM TRIS-HCl, pH 7.4, 120 mM NaCl, 5 mM KCl, 2 mM CaCl_2_, 1 mM MgCl_2_) without (total binding, TB; *n* = 3) or with co-incubation of 1 µM haloperidol (nonspecific binding, NB; *n* = 3) at room temperature for 60 min. The incubation was terminated by filtration via a Whatman^®^ glass microfibre filter (Grade GF/B, pre-incubated in freshly prepared polyethyleneimine (3%) at room temperature for 90 min), followed by quadruplicate washing with 50 mM TRIS-HCl, pH 7.4/4 °C using a semi-automated cell harvester (48-samples; Brandel, Gaithersburg, MD, USA). Filter-bound radioactivity was detected in terms of DPM/vial by liquid scintillation counting (Beckman LS 6500; Beckman Coulter Inc., Fullerton, CA, USA) of the isolated filters immersed for two hours in liquid scintillation cocktail (Ultima Gold; PerkinElmer LAS GmbH, Rodgau, Germany). Specific binding (SB) was calculated by SB (DPM/vial) = TB (DPM/vial) − NB (DPM/vial). The B_max_ and the K_D_ values were estimated by a nonlinear regression model (equation: one-site binding (hyperbola)) using GraphPad Prism, Version 4.1 (GraphPad Inc., La Jolla, CA, USA).

### 4.4. In Vitro Autoradiography on Human Glioblastoma Tissue

Cryosections of brain tumour tissue from three patients (Glioblastoma multiforme IV) were obtained using a microtome (MICROM HM560, Fisher Scientific GmbH, Schwerte, Germany), mounted on microscopy slides (SuperFrost, Thermo Scientific Menzel, Fisher Scientific GmbH, Schwerte, Germany), dried for ~2 h at room temperature, and stored at −25 °C until the autoradiography study. For the experiment, the slides were taken out from the freezer, the cryosections dried under a stream of cold air, and pre-incubated with incubation buffer (50 mM TRIS-HCl, pH 7.4, 120 mM NaCl, 5 mM KCl, 2 mM CaCl_2_, 1 mM MgCl_2_) at room temperature for 15 min. The pre-incubation solution was decanted, the slices dried again under a stream of cold air, and covered afterwards with the incubation solution ((*S*)-(−)-[^18^F]fluspidine, 197 kBq/mL incubation buffer = 4.5 nM at the time of incubation, without (total binding) or with co-incubation with 1 µM haloperidol to assess nonspecific binding). Incubation at room temperature was terminated after 60 min, the slides were washed two times in 50 mM TRIS-HCl, pH 7.4 at 4 °C, on ice for two minutes each followed by dipping in ice-cold demineralized water for 5 s and rapid drying under a stream of cold air. Afterwards, the slides were exposed to a phosphor imager plate (BAS-IP TR 2025, FujiFilm Corporation, Tokyo, Japan) along with standards obtained by pipetting and drying 1 µL of each concentration of a serial dilution of the radioligand solution on to a microscopic slide. The exposed phosphor-imaging plates were scanned using a high resolution scanner (HD-CR 35 Bio; Dürr NDT GmbH & Co. KG, Bietigheim-Bissingen, Germany) at a laser spot size of 12.5 µm (pixel size: 12.5 µm^2^) followed by two-dimensional analysis of the digitized images (AIDA 4.27; Elysia-raytest GmbH, Straubenhardt, Germany). The tracer distribution in the autoradiographic images obtained for total and nonspecific binding was compared by visual inspection and correlated with the histochemical staining (Nissl- and Hematoxiline-eosin staining) of the corresponding tissue sections.

### 4.5. In Vitro Autoradiography on Mice Brain-Bearing Glioblastoma

Cryosections of brains obtained from female athymic nude mice (Rj:NMRI-Foxn1 nu/nu) (10–12 weeks old, 25–38 g), were obtained as described above. The same protocol as in Section 4.3 was used. The incubation step was performed with 0.1–0.2 MBq/mL (*S*)-(−)-[^18^F]fluspidine in buffer for 60 min at room temperature. Nonspecific binding was determined in the presence of 10 µM of SA4503 (Tocris, Bio-Techne GmbH, Wiesbaden-Nordenstadt, Germany) or 100 µM to 10 nM of (*S*)-(−)-fluspidine, respectively. Developed autoradiographs were analysed in a phosphor imager (HD-CR 35; Dürr NDT GmbH & Co. KG, Bietigheim-Bissingen, Germany). The quantification was performed by using 2D-densitometric analysis (AIDA 2.31 software; raytest Isotopenmessgeräte GmbH, Straubenhardt, Germany). The B_max_ and the K_D_ values were estimated by a linear regression model (equation: one-site binding (hyperbola)) using GraphPad Prism, Version 4.1 (GraphPad Inc., La Jolla, CA, USA).

### 4.6. Immunohistochemistry

Tissues were cryopreserved by incubation in 2-Methylbutane at −25 °C (Merck, Germany). The brains were cut into coronal sections 10 µm thickness with cryostat (MICROM HM560, Fisher Scientific GmbH, Schwerte, Germany) and kept at −25 °C. Immunostaining was performed after fixation in PFA 4% for 20 min at 4 °C of the slides. Detection of the sig1R protein was performed by overnight incubation at 4 °C of the slides with the primary mouse monoclonal antibody (1:500 in blocking buffer 5% normal goat serum, B-5: sc-137075, Santa Cruz Biotechnology, Inc., Dallas, TX, USA). After washing with a solution of 1% BSA in PBS, the slides were incubated for 1 h at room temperature with the secondary polyclonal goat anti-mouse antibody (1:200 in dilution buffer 1% BSA, Alexa Fluor ^®^ 488; ab150117, Abcam, Berlin, Germany). A Hoechst counterstaining, 10 min at room temperature, was performed to visualize the nuclei of the cells (1:1000 in PBS, Hoechst 33258, Life Technologies, Carlsbad, Ca, USA). After a step of washing and drying, slides were cover up with mounting medium. (Aquapolymount, Polysciences Europe GmbH, Hirschberg an der Bergstrasse, Germany). Visualization of the slides was performed by fluorescence microscopy (Leica, DMi8, software Leica LASX, Leica Mikrosysteme Vertrieb GmbH, Wetzlar, Germany).

### 4.7. Animals and Orthotopic Brain Tumour Model

Female athymic nude mice (Rj:NMRI-Foxn1 nu/nu) were chosen for this study (Janvier labs, France). The mice were used for tumour implantation at the age of 8 weeks (26–30 g). During microsurgery mice were anesthetized with a mixture of air and isoflurane concentrate (1.5–2% depending on the breathing) under sterile conditions. The mice were placed into a Stoelting stereotactic frame (just for mouseTM, Stoelting Europe, Dublin, Ireland). A midline incision was done and a burr hole was drilled 0.5 mm anterior and 2.5 mm lateral to the bregma. 5 × 10^4^ U87-MG cells were suspended in 1 μL Hank’s Buffered Salt Solution (HBSS, 1X) and were injected 3.0 mm into the brain parenchyma with a flow of 0.1 μL/min using a 10 μL Hamilton syringe. After injection, the burr hole was filled with bonewax (Ethicon, US, LLC), the scalp incision sutured (Vicryl 6.0, Ethicon, US, LLC) and the surface antiseptically cleaned. Animal sacrifice was performed by induction of anesthesia with a mixture of air and isoflurane concentrate followed by cervical dislocation.

### 4.8. Small Animal PET/MR Imaging

For the time of the experiments, female CD-1 mice (*n* = 3; age: 10 weeks; weight: 30–35 g) or *nude* mice (*n* = 3; age: 10 weeks; weight: 25–30 g) (Janvier Labs, Le Genest-Saint-Isle, France) and one CD-1 sig1R-knockout mouse (*n* = 1; age: 10 weeks; weight: 27 g) (Envigo RMS, SARL, Bresso, Italy) were kept in a dedicated climatic chamber with free access to water and food under a 12:12h dark:light cycle at a constant temperature of 24/26 °C. The animals were anaesthetized (Anaesthesia Unit U-410, AgnTho’s, Lidingö, Sweden) with isoflurane (1.8%, 0.35 L/min) delivered in a 60% oxygen/40% air mixture (Gas Blender 100 Series, MCQ instruments, Rome, Italy) and maintained at 37 °C with a thermal bed system. (*S*)-(−)-[^18^F]fluspidine was injected into the lateral tail vein (control group: 3.5 ± 1.9 MBq, A_m_: 94 ± 7 GBq/µmol EOS; *h*sig1R-knockout mouse: 1.9 MBq; A_m_: 89 GBq/µmol EOS; tumour group: 5.7 ± 3.7 MBq; A_m_: 119 ± 41 GBq/µmol EOS) followed by a 60 min PET/MR scan (nanoScan^®^, Mediso, Hungary). Each PET image was corrected for random coincidences, dead time, scatter and attenuation (AC), based on a whole body (WB) MR scan. The list mode data were sorted into sonograms using a framing scheme of 12 × 10 s, 6 × 30 s, 5 × 300 s, 9 × 600 s. The reconstruction parameters for the list mode data are: 3D-ordered subset expectation maximization (OSEM), 4 iterations, 6 subsets, energy window: 400–600 keV, coincidence mode: 1–5, ring difference: 81. The mice were positioned prone in a special mouse bed (heated up to 37 °C), with the head fixed to a mouth piece for the anesthetic gas supply with isoflurane in 40% air and 60% oxygen. The animal head was positioned in the center of the field of view in order to benefit from the highest spatial resolution possible (spatial resolution center of the FOV: 900 µm). A dynamic PET scan of a duration of 60 min was performed followed by a T2 weighted sequence (Fast Spin Echo, TR/TE: 4377/88.5 ms, NEX: 4, FOV: 70 × 70 mm, matrix: 256 × 256, SI: 0.9 mm) and a T1 weighted sequence (Gradient Echo, TR/TE: 15/2.59 ms, NEX: 4, FOV: 60 × 60 mm, matrix: 160 × 160, slice thickness: 0.5 mm) for anatomical orientation and AC correction respectively. Image registration and evaluation of the region of interest (ROI) was done with PMOD (PMOD Technologies LLC, v. 3.9, Zurich, Switzerland). The respective brain regions were identified using the T2 weighted sequence and the tumour area and the contralateral area were delineated manually. The hypersignal due to the tumour in T2 weighted images was manually segmented and described as “tumour ROI”, and due to the compression of the contralateral side a fixed circled shape ROI was used to delineate the striatum avoiding nearby structure (cortex, ventricles, hypothalamus). The image-derived input function (IDIF) was extracted from a voxel of interest (VOI) segmented on the inferior vena cava (IVC). The IVC was identified using the first time frames showing the first passage of (*S*)-(−)-[^18^F]fluspidine bolus. An automatic algorithm from PMOD was used to identify the IVC signal avoiding heart and kidney area [76]. The activity data are expressed as mean standardized uptake value (SUV) of the overall ROI or as SUV ratio of the striatum ROI over the IDIF (SUVR). Data are presented as mean ± standard deviation (SD). Microsoft Excel was used to perform statistical tests. A parametric student *t*-test preceded by a Fischer test for variance were used to compare the groups with *p* < 0.05.

## 5. Conclusions

To conclude, we showed for the first time in an orthotopic GBM model, the U87-MG mouse model of glioblastoma and the suitability of a sig1R-targeting PET radioligand, (*S*)-(−)-[^18^F]fluspidine, to investigate the tumour-specific expression pattern of sig1R by in vivo imaging. Whether the inferior outcome in vivo in comparison to in vitro is caused by the physiological expression of sig1R in the healthy brain or by certain pathophysiological characteristics of the orthotopic mouse model of GBM, remains to be elucidated. Nevertheless, this first evaluation of the sig1R availability in an orthotopic in vivo model of brain tumour contributes to a better understanding of this model and suggests an expression of sig1R in the tumour periphery as found in other studies, which may be related to proliferation and invasiveness. In conclusion, the data obtained in the U87-MG mouse model of GBM along with the detection of sig1R in human GBM tissue for the first time by a PET radioligand, indicate not only the relevance of this target but also the suitability of (*S*)-(−)-[^18^F]fluspidine for sig1R-targeted cancer research and drug development.
